# Utility of the mini-mental state examination for assessing cognitive function in determining appropriate medication management strategies in patients with heart failure: a retrospective observational study

**DOI:** 10.1186/s40780-026-00567-1

**Published:** 2026-03-28

**Authors:** Naoya Ashikawa, Kana Inagaki, Yusuke Oka, Takashi Doi, Takayo Kawane, Yuko Sago

**Affiliations:** Department of Pharmacy, Toyohashi Heart Center, 21-1 Gobutori, Oyama-cho, Toyohashi, Japan

**Keywords:** Mini-Mental State Examination (MMSE), Heart failure, Medication adherence, Medication calendar, Medication envelopes

## Abstract

**Background:**

Patients with heart failure frequently experience recurrent hospitalizations, with poor medication adherence serving as a primary determinant of adverse clinical outcomes. Given the elevated prevalence of cognitive impairment among elderly patients with heart failure, the implementation of individualized medication management strategies is imperative. This study aimed to elucidate the association between Mini-Mental State Examination (MMSE) scores and the feasibility of various medication management strategies in hospitalized patients with heart failure.

**Methods:**

This retrospective observational study analyzed 148 patients with heart failure aged ≥ 65 years. Subjects were categorized into three groups based on their medication management at discharge: self-management via medication envelopes (press-through-package sheets or unit-dose packaging), self-management using a medication calendar, or nurse-assisted administration. Multivariate logistic regression and receiver operating characteristic (ROC) curve analyses were performed to identify predictors of self-management feasibility.

**Results:**

The MMSE score was the sole parameter showing significant differences across all pairwise comparisons between management groups. Multivariate analysis identified the MMSE score as an independent predictor for both self-management via envelopes and the necessity of adherence aids. For self-management utilizing medication envelopes, ROC analysis yielded an area under the curve (AUC) of 0.848, with an optimal cutoff threshold of ≥ 27 (sensitivity: 78.6%; specificity: 80.2%). For the broader feasibility of self-administration (using either envelopes or calendar), the AUC was 0.820, with a threshold of ≥ 24 (sensitivity: 83.7%; specificity: 67.9%). Additionally, patients of heart failure-related readmission associated with poor adherence occurred exclusively when established management protocols were discontinued post-discharge.

**Conclusions:**

MMSE scores may represent a valuable tool for predicting the successful establishment of medication management strategies. Specifically, scores below 27 suggest a need for adherence aids like medication calendar, while scores below 24 indicate a substantial requirement for external caregiver or nursing assistance to ensure therapeutic adherence.

## Background

Patients with heart failure frequently experience recurrent hospitalizations due to worsening symptoms, which are also associated with an increased risk of mortality. In Japan, over 250,000 patients with heart failure are hospitalized annually due to worsening symptoms, with an in-hospital mortality rate of approximately 8% [1]. Therefore, preventing such hospitalizations is critically important for this patient population. Poor medication adherence is an important risk factor for rehospitalization in this population [2], making it essential to maintain good medication adherence to improve patient outcomes. Previous research indicates that interventions targeting improved medication adherence can reduce both mortality and rehospitalization rates among patients with heart failure [3]. Consequently, it is imperative to establish a medication management strategy that is suitable for each patient. However, since many patients of heart failure are elderly [4], they are known to have a high risk of developing dementia [5]. Furthermore, cognitive impairment is recognized as a major factor contributing to poor medication adherence [6].

One of the most widely used diagnostic tools for assessing cognitive function is the Mini-Mental State Examination (MMSE), developed by Folstein et al. in 1975 [7]. The MMSE consists of 11 questions assessing disorientation, memory, calculation, verbal, and graphical abilities. The test is scored on a 30-point scale, with a score of 26 or below indicating mild cognitive impairment and a score of 23 or below indicating a strong suspicion of dementia. The association between cognitive function as assessed by the MMSE and the ability to self-administer medication has previously been examined. In studies of elderly patients, a MMSE score of 23 or below was independently associated with poor medication adherence in their multivariate logistic regression analysis [8]. Furthermore, when medication management was carried out using medication envelopes, more than half of the patients with MMSE scores of 26 or below made medication errors, and all patients with scores of 21 or below made such errors [9].

In contemporary healthcare settings, encountering patients with MMSE scores of 26 or below is increasingly common due to the aging population. Moreover, it is virtually infeasible to expect family members or caregivers to manage medications for all such patients, particularly given the rising incidence of cognitively impaired individuals living alone or in elderly-only households [10]. On the other hand, for these patients, fostering medication self-management to enhance their awareness of their illness is as vital as maintaining good medication adherence. One potential solution to these challenges is the use of a medication calendar, a type of adherence aid that can effectively indicate whether a medication has been taken. Previous studies [8] have suggested that not utilizing medication calendar is associated with poor medication adherence.

However, few studies have examined the relationship between cognitive function and medication management strategies in patients with heart failure. Moreover, no prior research has specifically investigated the association between the need for adherence aids, such as medication calendar, and MMSE scores. Patients with heart failure often have a higher burden of comorbidities [11], necessitating complex regimens with frequent dosing. Despite these circumstances, ensuring good medication adherence is crucial for patients with heart failure in order to improve their clinical prognosis. In this context, we evaluated the utility of MMSE score in identifying patient subgroups for whom adherence aids, primarily medication calendar, are essential for maintaining good medication adherence. Furthermore, to facilitate the early screening of individuals requiring medication support, we investigated whether MMSE score could serve as a predictor for identifying patients incapable of self-management, even with the implementation of adherence aids. Investigating these associations could provide valuable insights for designing tailored medication management strategies. Therefore, this study aimed to examine the correlation between MMSE scores and medication management strategies in patients with heart failure, and to evaluate the utility of the MMSE as a predictive tool for tailoring these interventions.

## Methods

### Subjects

This retrospective observational study included patients aged 65 years or older who were diagnosed with heart failure and admitted to the Toyohashi Heart Center between October 2019 and March 2020. Patients with heart failure caused by onset of acute myocardial infarction were also included in this study. Exclusion criteria included death during hospitalization, cognitively impaired patients for whom the MMSE could not be performed, patients whose hospital stay was too short to establish an appropriate medication management strategy, and patients with unknown outcomes for 6 months after discharge. During hospitalization, a multidisciplinary team provided lifestyle guidance to all patients and their families to prevent worsening heart failure.

### Assessment of cognition and medication management

The MMSE was conducted by nurses as soon as possible after admission, using the MMSE-J Revised Japanese Edition (Nihon Bunka Kagakusha Co., Ltd.). The assessment was administered in accordance with an internally developed manual to ensure the standardization of the procedure.

The initial settings for medication management during hospitalization were based on findings from previous study [9]. Patients with an MMSE score of 27 or above and good medication adherence maintained their pre-admission medication management strategies. Conversely, for patients exhibiting poor medication adherence, those previously managed with PTP sheets were transitioned to unit-dose packaging using medication envelopes, whereas those already receiving unit-dose packaging were switched to management using a medication calendar. For patients with MMSE scores between 22 and 26, medication management using a medication calendar was introduced. Patients with MMSE scores of 21 or below were at high risk of errors when managing their medication, so they started with medication management using nurse assistance. Medication adherence was assessed as good when the pharmacist checked the drugs brought by the patient on admission and predicted the medication adherence rate to be 80% or more based on the prescription date, duration, and the quantity of residual medication, and as poor when it was less than 80%. The pharmacist or nurse was responsible for instructing patients on the newly introduced medication management strategy. Patients who were introduced to medication management using a medication calendar provided with one that could distribute medications three times a day for a week. If the medication was taken more than three times a day, only that medication was managed in medication envelopes or multiple medication calendars were used. Medication distribution to the medication calendar was carried out by the patient or by a healthcare professional at the hospital. If medication was administered using patient-owned adherence aids (medication calendar or pill organizer), this was handled as administration using a medication calendar. During hospitalization, the transition from medication management using nurse assistance to self-administration was made as much as possible, and the use of the medication calendar was discontinued if it was not necessary for administration. To identify medication errors in patients, in cases where press-through-package sheets or unit-dose packaging were managed using medication envelopes, medications were checked three times a week. When the medication calendar was used, the medications were checked by the pharmacist or nurse at each administration due to the high risk of medication errors. If two or more medication errors occurred during management using medication envelopes, management was transitioned to using medication calendar. Additionally, if two or more medication errors occurred during management using medication calendar, management was transitioned to nurse assistance.

### Data collection

We collected data on demographics, medication management strategies, MMSE scores, medications, comorbidities, laboratory tests, and echocardiograms at discharge. Regarding the number of prescribed drugs and the daily dosing frequency, medications for which self-adjustment was permitted, such as laxatives and hypnotics, were excluded from the count. To assess the validity of the prescribed medication management strategies, we also investigated whether patients exhibited poor medication adherence among those re-hospitalized within six months of discharge due to worsening heart failure symptoms.

### Statistical analysis

Continuous variables are expressed as the median with interquartile range (IQR). The significance of differences in continuous variables was determined using the Kruskal-Wallis test. The significance of differences between three groups was assessed using the Steel-Dwass test. Categorical variables were reported as counts and percentages. The significance of differences between each group compared using the χ2 test. To eliminate confounding factors, a logistic regression analysis was performed using four covariates that had been reported in previous studies to be associated with medication adherence: age [12], MMSE score [9], daily dosing frequency [13], and number of prescribed drugs [14]. Regard these covariates, we assessed the presence of multicollinearity by calculating the variance inflation factor (VIF). Odds ratios (OR) and 95% confidence intervals (CI) were calculated for two outcomes: whether medication self-administration was possible and whether a medication calendar was required in cases where medication self-administration was possible. Optimal cutoff values were determined by constructing a receiver operating characteristic (ROC) curve and applying the Youden Index method. For all tests, a difference was considered significant if *p* < 0.05. Statistical analyses were performed using SPSS Statistics Version 22.0 (IBM, Armonk, USA).

## Results

**Fig. 1:**
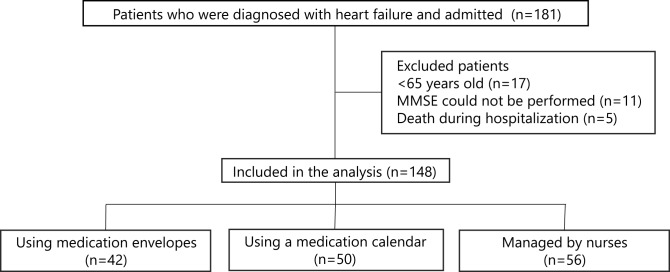
Flow chart of study patient selection and medication management strategies at discharge. Abbreviations: MMSE, Mini-Mental State Examination

Between October 2019 and March 2020, 181 patients were hospitalized at Toyohashi Heart Center with a diagnosis of heart failure. Of this initial cohort, 33 individuals were excluded for meeting the predefined criteria, resulting in a final study population of 148 patients (Fig. 1). Table 1 summarizes the baseline characteristics of the participants, categorized by their medication management strategy at discharge. The median (IQR) length of hospitalization for all patients was 16(11–24) days. Among the enrolled patients, 42 self-managed their medication using medication envelopes, 50 self-managed their medication using a medication calendar, and 56 had their medication managed by nurses. For patients using medication envelopes, the analysis combined patients who managed their medications with PTP sheets (*n* = 16) and those who used unit-dose packaging (*n* = 26). Although significant differences were identified among several parameters, the MMSE score was the only parameter that exhibited a significant difference not only among the three groups but also in all pairwise comparisons. Specifically, the median (IQR) MMSE scores were 27 (27–29) for patients using medication envelopes, 26 (23–27) for those using a medication calendar, and 20 (17–25) for individuals whose medications were managed by nurses. Table 2 summarizes the medication management strategies implemented at the time of admission and discharge. Among patients whose medication was managed by caregivers upon admission, the majority required similar strategy at the time of discharge. Conversely, of the 103 patients utilizing medication envelopes at admission, 39 continued with the same strategy at discharge, whereas the remaining individuals transitioned to management using medication calendar or nurse assistance.

**Table 1:** Baseline characteristics

	Medication management strategy at discharge				
	All	Using medication envelopes	Using medication calendar	Nurse assistance	p value
n	148	42	50	56	
Age	83 [78–87]	79 [72–82]	83 [79–87]*	85 [82–89]*	<0.001
Body weight (kg)	49.0 [42.5–57.8]	52.4 [48.6–61.7]	47.4 [42.0–58.6]	47.5 [40.9–53.9]*	0.007
MMSE score	26 [21–27]	27 [27–29]	26 [23–27]*	20 [17–25]*#	<0.001
≤26	94 (63.5)	9 (21.4)	36 (72.0)	49 (87.5)	<0.001
≤23	53 (35.8)	2 (4.8)	13 (26.0)	38 (67.9)	<0.001
Daily dose frequency	2 [2, 3]	2 [1–3]	2 [1–3]	3 [2, 3]*	0.024
Number of prescribed drugs	9 [7–11]	9 [6–11]	10 [8–11]	10 [7–11]	0.783
Length of hospitalization (day)	16 [11-24]	14 [10-18]	19 [12-24]	16 [10-26]	0.058
LVEF (%)	52.0 [39.1–60.8]	44.6 [35.8–57.2]	49.5 [37.9–58.8]	58.4 [46.0–64.3]	0.003
HFrEF	38 (25.7)	15 (35.7)	14 (28.0)	9 (16.1)	0.079
NYHA III/IV	8 (5.4)	1 (2.4)	1 (2.0)	6 (10.7)	0.083
Serum creatinine (mg/dL)	1.3 [0.9–1.8]	1.2 [1.0–1.8]	1.2 [0.9–1.6]	1.5 [1.0–2.2]	0.163
Creatinine clearance (mL/min)	27.4 [17.9–42.9]	33.3 [22.1–46.5]	31.5 [22.8–43.2]	21.0 [13.5–30.6]*#	
Blood Hb (g/dL)	11.4 [9.9–12.6]	11.6 [9.8–12.7]	11.4 [10.5–12.7]	10.5 [9.3–12.2]	0.055
Serum BNP (pg/mL)	403 [182–747]	402 [206–681]	412 [163–670]	396 [173–817]	0.748
Comorbidity					
Hypertension	84 (56.8)	23 (54.8)	26 (52.0)	35 (62.5)	0.527
Dyslipidemia	58 (39.2)	21 (50.0)	23 (46.0)	14 (25.0)	0.021
Acute myocardial infarction	16 (10.8)	5 (11.9)	9 (18.0)	2 (3.6)	0.056
Ischemic heart disease	62 (41.9)	23 (54.8)	23 (46.0)	16 (28.6)	0.026
Valvular heart disease	42 (28.4)	6 (14.3)	15 (30.0)	21 (37.5)	0.04
Atrial fibrillation	69 (46.6)	18 (42.9)	27 (54.0)	24 (42.9)	0.438
Diabetes	96 (64.9)	29 (69.0)	34 (68.0)	33 (58.9)	0.496
Medication for heart failure					
Diuretics	128 (86.5)	36 (85.7)	44 (88.0)	48 (85.7)	0.929
ACE inhibitor/ARB	97 (65.5)	33 (78.6)	35 (70.0)	29 (51.8)	0.016
MRA	66 (44.6)	16 (38.1)	29 (58.0)	21 (37.5)	0.064
βbloclker	108 (73.0)	35 (83.3)	36 (72.0)	37 (66.1)	0.16
SGLT2 inhibitor	13 (8.8)	8 (19.0)	3 (6.0)	2 (3.6)	0.019

Values are n(%) or median [IQR]

*; significant difference for group of using medication envelopes (*p* < 0.05)

#; significant difference for group of using medication calender (*p* < 0.0)

**Table 2:** Medication management strategies at the time of admission and discharge

		At discharge		
		Using medication envelopes	Using medication calendar	Nurse assistance
At admission	Using medication envelopes (*n* = 103)	39	41	23
	Using adherence aids (*n* = 6)	0	3	3
	Assistance of caregiver (*n* = 32)	1	1	30
	No medication (*n* = 7)	2	5	0

### Factors associated with medication management strategies

**Fig. 2:**
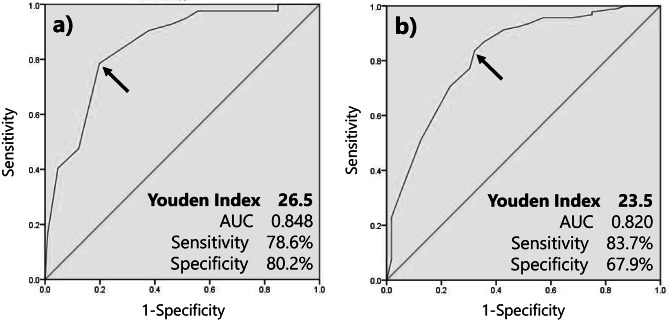
The ROC curve for the association between medication management strategies and MMSE scores. **a**) Feasibility of medication self-management using medication envelopes. **b**) Feasibility of medication self-management using medication envelopes or a medication calendar. Abbreviations: MMSE, Mini-Mental State Examination; ROC, receiver operating characteristic

To examine factors influencing the feasibility of medication self-management using medication envelopes, a multivariate logistic regression analysis was conducted with age, MMSE score, daily dosing frequency, and the number of prescribed drugs as covariates (Table 3). The VIF values for the four covariates were 1.147, 1.151, 1.211, and 1.220, respectively; these results indicate that no significant multicollinearity was present within the model. The analysis identified age (OR 0.927, 95% CI 0.867–0.990) and MMSE score (OR 1.519, 95% CI 1.235–1.870) as significant factors. Similarly, to examine factors influencing the feasibility of medication self-management using either a medication calendar or medication envelopes, a multivariate logistic regression analysis was conducted with the same four covariates (Table 4). This analysis identified MMSE score (OR 1.341, 95% CI 1.200–1.499) and the daily dose frequency (OR 0.567, 95% CI 0.375–0.857) as significant factors. Since the MMSE score was a common associated factor for both analyses, ROC curves generated and cutoff values determined. The area under the curve (AUC) for medication self-management using medication envelopes was 0.848, with a Youden Index of 26.5 (Fig. 2a). Accordingly, when the cutoff value for acceptable medication self-management was set at 27 points or higher, the sensitivity and specificity were 78.6% and 80.2%, respectively. Similarly, the AUC for medication self-management using medication envelopes or a medication calendar was 0.820, with a Youden Index of 23.5 (Fig. 2b). When the cutoff value was set at 24 points or higher, the sensitivity and specificity were 83.7% and 67.9%, respectively.

**Table 3:** Multivariate logistic regression analysis of factors influencing the feasibility of medication self-management using medication envelopes

Factor	Odds Ratio	95% CI
Age	0.927	0.867–0.990
MMSE score	1.519	1.235–1.870
Daily dose frequency	0.773	0.480–1.245
Number of prescribed drugs	0.92	0.786–1.078

**Table 4:** Multivariate logistic regression analysis of factors influencing the feasibility of medication self-management

Factor	Odds Ratio	95％CI
Age	0.961	0.900–1.027
MMSE score	1.341	1.200–1.499
Daily dose frequency	0.567	0.375–0.857
Number of prescribed drugs	0.972	0.837–1.128

### Re-hospitalization and poor adherence to medication within 6 months of discharge

Re-hospitalization due to worsening heart failure symptoms within 6 months of discharge occurred in 32 patients (21.6%). Three (2.0%) of these patients were non-adherent to their medication at the time of re-hospitalization. None of the three non-adherent patients continued with the medication management strategy established during their hospitalization. Specifically, one patient who had initially adopted medication management using a medication calendar was instead using medication envelopes, while two patients who had introduced caregiver-assisted management were actually managing their medication independently.

## Discussion

This study examined the association between medication management strategies for maintaining good adherence in patients with heart failure and their scores on the MMSE, a standard instrument for cognitive assessment. The findings suggest that the MMSE score may be a robust predictor of a patient’s capacity for medication self-management and the necessity of utilizing adherence aids, such as a medication calendar. Regarding the eligibility age for this study, individuals aged 65 and older are entitled to the Community-Based Integrated Care System in Japan. For this reason, our institution routinely conducts the MMSE as part of the patient information provided to the Community-Based Integrated Support Center. Furthermore, clinical registry of patients of heart failure in Japan report an average age of approximately 80 years [10]. Therefore, we considered that research targeting elderly patients would be applicable to the majority patients of heart failure and conducted this study with patients aged 65 and older.

To date, conventional methods for evaluating medication adherence include the Morisky Medication Adherence Scale (MMAS), developed by Morisky et al. in 1986 [15], and various institution-specific assessment tools. However, for elderly patients with heart failure, who exhibit a high prevalence of comorbid dementia, it is often difficult to accurately report their medication adherence status. In the present study, the MMSE was employed to assess cognitive function in place of the revised Hasegawa’s Dementia Scale [16]. The rationale for this selection is that the MMSE incorporates tasks that involve following illustrated instructions and performing manual operations, rendering it a more suitable instrument for evaluating the capacity for medication self-management. Additionally, the MMSE is characterized by its ease of administration, typically requiring approximately 10 minutes to complete. Among the study participants, 53 individuals (35.8%) scored 23 points or below on the MMSE, indicating suspected dementia. When including those scoring 26 points or below, which indicates mild cognitive impairment, the number increased to 94 individuals (63.5%). Furthermore, this study excluded 11 patients with markedly impaired cognitive function who were unable to perform the MMSE, suggesting that cognitive function cannot be ignored when considering medication management strategies for elderly patients of heart failure.

Multivariate analysis identified the MMSE score as the sole independent factor significantly associated with the feasibility of self-management utilizing medication envelopes, as well as the feasibility of self-administration using either envelopes or medication calendar. Regarding the feasibility of self-management utilizing medication envelopes, the ROC curve analysis yielded an AUC of 0.848, indicating a moderate predictive accuracy for the MMSE score. The sensitivity and specificity for predicting self-management capability at a cut-off threshold of 27 were 78.6% and 80.2%, respectively. These findings suggest that the MMSE score possesses predictive utility for determining the feasibility of self- management using medication envelopes. This cut-off threshold is consistent with the findings previously reported by Miura et al. [9]. Nevertheless, even for elderly patients with high MMSE scores, the proactive adoption of adherence aids, such as medication calendar, may be warranted to further optimize management. Regarding the feasibility of self-administration utilizing either medication envelopes or calendar, the ROC curve analysis yielded an AUC of 0.820, indicating a moderate predictive accuracy for the MMSE score. The sensitivity and specificity for predicting self-management capability at a cut-off threshold of 24 were 83.7% and 67.9%, respectively. These findings suggest that the MMSE score possesses predictive utility for determining the feasibility of medication self-management using these tools. This cut-off threshold is consistent with the findings previously reported by Okuno et al. [8]. Furthermore, the use of adherence aids may enable medication self-management even among patients with mild cognitive impairment who were unable to manage their medication using medication envelopes. Although, the cut-off threshold identified in this study was higher than those reported in previous literatures [9]. This discrepancy may be attributed to the complexity of medication regimens; while prior studies did not provide specific data on daily dosing frequency, heart failure patients typically present with multiple comorbidities [11]. Consequently, it is plausible that the daily dosing frequency was higher in our cohort than in previous study populations [9]. Therefore, simplifying medication regimens by reducing dosing frequency, a factor identified as influential in this study, may expand the proportion of patients capable of medication self-management.

In summary, the utilization of MMSE scores is suggested to be not only efficient but also facilitates the identification of patients who are likely to encounter difficulties with medication self-management. This approach facilitates the preemptive determination of requirements for adherence aids, thereby potentially reducing the incidence of inpatient medication errors. Furthermore, it enables the identification of patients at high risk for poor adherence and clarifies the necessary medication support prior to discharge. In recent years, the prevalence of cognitively impaired individuals living alone or in elderly-only households has been increasing [10]. Consequently, initiatives designed to facilitate medication self-management to the greatest extent possible through the utilize of adherence aids such as a medication calendar are of growing importance. In the present study, the number of prescribed medications was not identified as a significant factor in either analysis; this is likely attributable to the widespread implementation of unit-dose packaging for most patients, which mitigated the potential influence of polypharmacy.

This study has several limitations. First, this study is retrospective design and relatively small sample size. Second, the possibility that the initial medication management strategies, guided by MMSE score, influenced the final strategies at discharge cannot be entirely excluded. However, given that management strategies were appropriately titrated during hospitalization and that all cases of heart failure readmission within six months due to poor adherence involved the discontinuation of established protocols, it is reasonable to infer that the strategies established during the inpatient period were clinically appropriate. Third, this study utilized only the MMSE score obtained at admission, and therefore may not fully reflect fluctuations in cognitive function during hospitalization. However, the medication management method at discharge was determined after evaluating the patient’s management capabilities during the hospitalization period, and we consider this reflects their actual cognitive function. Finally, the evaluation of medication adherence at the time of admission is based solely on the prescription date, duration, and the quantity of residual medication. Consequently, the potential for patients to have omitted bringing their remaining medication or to have disposed of it cannot be entirely ruled out.

## Conclusions

MMSE scores may represent a valuable tool for predicting the successful establishment of medication management strategies, such as self-administration via medication envelopes or calendar, in hospitalized patients with heart failure.

## Data Availability

The deidentified participant data will not be shared because the patients’ consent has not been obtained.

## Abbreviations

AUC: Area under the curve
CI: Confidence interval
IQR: Interquartile range
MMAS: Morisky Medication Adherence Scale
MMSE: Mini-Mental State Examination
OR: Odds ratio
ROC: Receiver operating characteristic

## References

[1] Nishi M, Miyamoto Y, Iwanaga Y, Kanaoka K, Sumita Y, Ishihara M, et al. Hospitalized patients, treatments, and quality of care for cardiovascular diseases in Japan - outline of the nationwide JROAD investigation. Circ J. 2024 Nov 19. Available from: 10.1253/circj.CJ-24-0704. 10.1253/circj.CJ-24-0704 39566969

[2] Tsuchihashi M, Tsutsui H, Kodama K, Kasagi F, Takeshita A. Clinical characteristics and prognosis of hospitalized patients with congestive heart failure-a study in Fukuoka, Japan. Jpn Circ J. 2020;64(12):953–59. 10.1253/jcj.64.953 11194290

[3] Ruppar TM, Cooper PS, Mehr DR, Delgado JM, Dunbar-Jacob JM. Medication adherence interventions improve heart failure mortality and readmission rates: systematic review and meta-analysis of controlled trials. J Am Heart Assoc. 2016;5(6):e002606. 10.1161/JAHA.115.002606 PMC4937243 27317347

[4] Sato Y, Kuragaichi T, Nakayama H, Hotta K, Nishimoto Y, Kato T, et al. Developing multidisciplinary management of heart failure in the super-aging society of Japan. Circ J. 2023;88(1):2–9. 36567108 10.1253/circj.CJ-22-0675

[5] Qiu C, Winblad B, Marengoni A, Klarin I, Fastbom J, Fratiglioni L. Heart failure and risk of dementia and Alzheimer disease: a population-based cohort study. Arch Intern Med. 2006;166(9):1003–08. 16682574 10.1001/archinte.166.9.1003

[6] Hawkins LA, Kilian S, Firek A, Kashner TM, Firek CJ, Silvet H. Cognitive impairment and medication adherence in outpatients with heart failure. Heart Lung. 2012;41(6):572–82. 22784869 10.1016/j.hrtlng.2012.06.001

[7] Folstein MF, Folstein SE, McHugh PR. “Mini-mental state”: a practical method for grading the cognitive state of patients for the clinician. J Psychiatr Res. 1975;12(3):189–98. 1202204 10.1016/0022-3956(75)90026-6

[8] Okuno J, Yanagi H, Tomura S. Is cognitive impairment a risk factor for poor compliance among Japanese elderly in the community? Eur J Clin Pharmacol. 2001;57(8):589–94. 11758637 10.1007/s002280100347

[9] Miura M, Kakei M, Iwasawa S, Morii T, Miura T, Sasaki H, et al. Assessment of compliance for oral medicines with MMSE, Mini-Mental State Examination, in hospitalized elderly patients. Yakugaku Zasshi. 2007;127(10):1731–38. 17917431 10.1248/yakushi.127.1731

[10] Yaku H, Ozasa N, Morimoto T, Inuzuka Y, Tamaki Y, Yamamoto E, et al. KCHF study investigators. Demographics, management, and In-hospital outcome of hospitalized acute heart failure syndrome patients in contemporary real clinical practice in Japan - observations from the prospective, multicenter kyoto congestive heart failure (KCHF) registry. Circ J. 2018;82(11):2811–19. 30259898 10.1253/circj.CJ-17-1386

[11] Zhang L, Ono Y, Qiao Q, Nagai T. Trends in heart failure prevalence in Japan 2014-2019. A report from healthcare administration databases. ESC Heart Fail. 2023;10(3):1996–2009. 37016908 10.1002/ehf2.14321 PMC10192231

[12] Garred CH, Zahir D, Butt JH, Ravn PB, Bruhn J, Gislason GH, et al. Adherence and discontinuation of optimal heart failure therapies according to age: a danish nationwide study. J Am Heart Assoc. 2022;11(19):e026187. 10.1161/JAHA.122.026187 PMC9673698 36172925

[13] Laliberté F, Nelson WW, Lefebvre P, Schein JR, Rondeau-Leclaire J, Duh MS. Impact of daily dosing frequency on adherence to chronic medications among nonvalvular atrial fibrillation patients. Adv Ther. 2012;29(8):675–90. 22898791 10.1007/s12325-012-0040-x

[14] Knafl GJ, Riegel B. What puts heart failure patients at risk for poor medication adherence? Patient Prefer Adherence. 2014;17(8):1007–18. 10.2147/PPA.S64593 PMC4109641 25114512

[15] Morisky DE, Green LW, Levine DM. Concurrent and predictive validity of a self-reported measure of medication adherence. Med Care. 1986;24(1):67–74. 3945130 10.1097/00005650-198601000-00007

[16] Imai Y, Hasegawa K. The revised Hasegawa’S dementia scale (HDS-R) -evaluation of its usefulness as a screening test for dementia. JHKC Psych. 1994;4:20–24.

